# Narrative therapy and family therapy in genetic counseling: A scoping review

**DOI:** 10.1002/jgc4.1938

**Published:** 2024-06-20

**Authors:** Aimee Dane, Jennifer Berkman, Emily DeBortoli, Courtney K. Wallingford, Tatiane Yanes, Aideen McInerney‐Leo

**Affiliations:** ^1^ Cardiology Department Prince Charles Hospital Brisbane Queensland Australia; ^2^ Dermatology Research Centre, Frazer Institute University of Queensland Brisbane Queensland Australia

**Keywords:** family therapy, genetic counseling, narrative therapy, psychotherapeutic, review

## Abstract

Genetic counseling facilitates psychological and social adaptation in clients and families. Two psychotherapeutic approaches, narrative and family therapy foster client adaptation to adverse situations and may enhance the genetic counseling process. This scoping review aimed to describe the applications of narrative therapy and family therapy in genetic counseling, and to document the actual and perceived value of these approaches in a genetic counseling setting. Nine original research articles and six commentary articles met the study inclusion criteria. Original articles reported on positive client attitudes when these approaches were applied to hereditary cancer and Huntington disease settings. Five studies applied either approach in group sessions, where safety was key to positive outcomes, including sharing lived experiences and coping strategies. Balanced utilization of structured and open elements in group sessions maximized a sense of control, while also allowing for opportunity to self‐disclose. Narrative therapy interventions were time efficient and were reported to foster connection with others and shape a new adaptive narrative centered around strengths. Family therapy approaches, based on experiential family therapy, the intersystem model, object relations family therapy, and the social ecology model, required a greater time commitment, but promoted disclosure of complex feelings and diffused tension. Family therapy genogram tools were feasible in practice, easy to implement, and effective at identifying communication barriers. Commentary articles highlighted the alignment of both approaches with genetic counseling goals and their potential value in practice. Utilization of psychotherapeutic approaches can improve counselors' ability to shape sessions, enhance insight and optimize efficacy, and flexibility in moving between models can maximize impact. This review highlights the paucity of studies investigating the efficacy of these psychotherapeutic approaches in the genetic counseling context and the need for more outcomes‐based research on the utilization of narrative or family therapy in genetic counseling practice.

**What is known about this topic**

The value of psychotherapeutic approaches in counseling has long been recognized but little is known about their utilization and impact in genetic counseling.

**What this paper adds to the topic**

This review summarizes the limited literature on the use of narrative therapy and family therapy in genetic counseling and showed that both approaches provided clients with new insights and allowed for a greater sense of control. Although positive impacts in research settings were captured qualitatively (sharing lived experiences and coping strategies in a safe, supportive environment), and quantitatively (low generalized anxiety and depression post‐session), no studies applied either approach in a standard genetic counseling consultation.

## 1 INTRODUCTION

The term “genetic counseling” was coined in 1948 (Reed, 1974), but was more precisely described by Seymour Kessler in 1979 as a “*kind of psychotherapeutic encounter*” (Kessler, 1979). This description aligns with the 2006 National Society of Genetic Counselors' definition of genetic counseling (National Society of Genetic Counselors' Definition Task et al., 2006) and the 2007 model of practice for genetic counseling, the Reciprocal Engagement Model (REM) (Veach et al., 2007), which emphasize the importance of attending to the psychological and social implications of conditions for individuals and families. Furthermore, while genetic counseling practice is continually evolving, Resta (2019) maintains that ethically, genetics services must demonstrate that they “*help to improve psychological, social, adaptational, and medical outcomes*” (Resta, 2019). As psychotherapeutic approaches enhance psychological and social adaptation (Christopher, 1986; Mejías et al., 2020), it is important for genetic counselors to be comfortable utilizing them in practice (Austin et al., 2014).

There are a number of psychotherapeutic approaches and theories which are relevant to genetic counseling practice including the well‐described, client‐centered counseling (Rogers, 1951) and more rarely reported, cognitive‐behavior therapy and fuzzy trace theories (Biesecker et al., 2017) and acceptance and commitment therapy (Broley, 2013). Two psychotherapeutic approaches which are frequently mentioned and salient to the field, given their potential to facilitate adaptation, are narrative therapy and family therapy. Narrative therapy focuses on story development and the meaning that is made from these stories. It seeks to separate people from their problems in order to facilitate positive change (Denborough, 2014). Family therapy focuses on understanding family beliefs, contexts, patterns of communication and behavior to help resolve the presenting problem (Carr, 2016). These two approaches have distinct goals but can be utilized together to elicit the meaning made from different family members' stories about a particular problem to facilitate understanding and resolution (Freedman, 2014). It can be hypothesized that each approach would translate well to genetic counseling, but they have received less attention than relationship‐based approaches such as Rogers' client‐centered approach (Rogers, 1951). Their effectiveness has been well documented in other settings such as in assisting adolescents with autism (narrative therapy) (Cashin et al., 2013) and recovery from anorexia nervosa (family therapy) (Eisler et al., 1997) but evidence of their use in genetic counseling is limited. Narrative therapy and family therapy may each provide advantages when addressing the unique psychological and social complexities which accompany genetic conditions and genetic information. These complexities include the shared nature of genetic information and the potential for emotionally complex and nuanced decision‐making (Jamal et al., 2020). Both narrative therapy and family therapy approaches are applicable to the individual and group psychotherapeutic setting.

Narrative therapy was developed by Michael White and David Epston as a collaborative approach to counseling and community work that is centered on the myriad of stories within one's life that shape lived experience (White & Epston, 1990). It is informed by family therapy and other theories and philosophies including Foucault's theory of power and knowledge (Schirato & Danaher, 2020). Important influences also included post‐modernism, which challenges existing world views, and allows the consideration and acceptance of many truths (Ellaway, 2020), and social constructivism which emphasizes the social context in the development of ideas and attitudes (Rees et al., 2020). These theories question the societal standards or truths by which individuals may judge themselves (Doan, 1997). Narrative therapy encourages the individual to consider multiple stories which underlie their lived experience and identify the relevant cultural and historical context which may be impacting their stories (Denborough, 2012). Using a narrative therapy approach, clients are viewed as experts of their lives, possessing the skills, experience, and values needed to adapt to adversity (Morgan, 2000; White & Epston, 1990). This therapy seeks to “double listen” to (a) the client's narrative and (b) their corresponding responses to the problem (MacLeod et al., 2018; White, 2004). Externalizing conversations are employed to craft language that separates the problem from the person (Denborough, 2014; Morgan, 2000). For example, a client's loneliness can be described as *“the loneliness you feel”* rather than *“you are lonely*.” Separating people from the problems they face through language creates space for reconsidering how one relates to their problems and facilitates identification of skills needed to overcome or adapt (MacLeod et al., 2021; White, 2004). Of note, narrative therapy is distinct from narrative medicine which is informed by many frameworks including narrative therapy. Narrative medicine encourages clinicians to listen, interpret and be moved by patients' stories of illness but was excluded from this review because it does not employ the narrative therapy techniques described above (Charon et al., 2017).

Family therapy or family systems therapy, developed from Bowen's family systems theory, views the individual within the context of a family's emotional unit. It posits that emotional patterns of functioning are repeated over generations (Bowen, 1978; Brown, 1999). Central concepts include differentiation of self and fusion with one or more family members. (Bowen, 1978; Brown, 1999). “Fusion” describes an individual's prioritization of family or relationship harmony over their own needs (Brown, 1999) (p. 95). Family therapy seeks a balance between family togetherness and healthy individuality (Bowen, 1978). It goes beyond the presenting problem to uncover the multiple factors contributing to its development and the potential resolutions (Brown, 1999; Carr, 2016). Tools such as the genogram, which is a diagram constructed within a session to illustrate family relationships and medical history, are used in family therapy to facilitate understanding of past relational patterns. The process of constructing the genogram can create a sense of distance from the client's current struggle, fostering insight into their own contributions to family functioning and the potential for behavior change (Brown, 1999, p. 97). Typically, techniques target family behaviors and beliefs or address systemic factors (Carr, 2016).

Although narrative therapy and family therapy are widely used in psychology practice and offer potential benefits for genetic counseling practice, little is known about the perceived value of their utilization in genetic counseling. This scoping review identified original research and commentary articles and aimed to describe the applications of narrative therapy and family therapy in genetic counseling, and to document the actual, and perceived value of these approaches in a genetic counseling setting.

## 2 METHODS

A scoping review was conducted according to the recommendations of Pollock et al. (2023) and the PRISMA (Preferred Reporting Items for Systematic Reviews and Meta‐Analyses) guidelines for Scoping Reviews (Tricco et al., 2018).

### 2.1 Search strategy

Relevant records were identified through searching databases CINAHL, PsycINFO, and PubMed using the following search terms: “genetic counsel* AND narrative OR family therapy OR, family theory OR, family system OR, multi‐family group OR, colored eco‐genetic relationship map (CEGRM)”. See Table S1 for search strings. Articles identified from reference and citation searches of articles meeting inclusion criteria were also included.

### 2.2 Inclusion and exclusion criteria

We aimed to capture original research or commentary articles that assessed implementation of narrative therapeutic or family therapeutic approaches in the genetic counseling context. The search was limited to articles published in English, and between January 1997 and January 2024. Commentary pieces were included to capture the range of attitudes and perceived value of these approaches in genetic counseling. Excluded articles included those describing narrative medicine and studies which quantitatively evaluated elements relevant to narrative and family therapy, in the absence of a counseling intervention.

### 2.3 Data extraction and analysis

**FIGURE 1:**
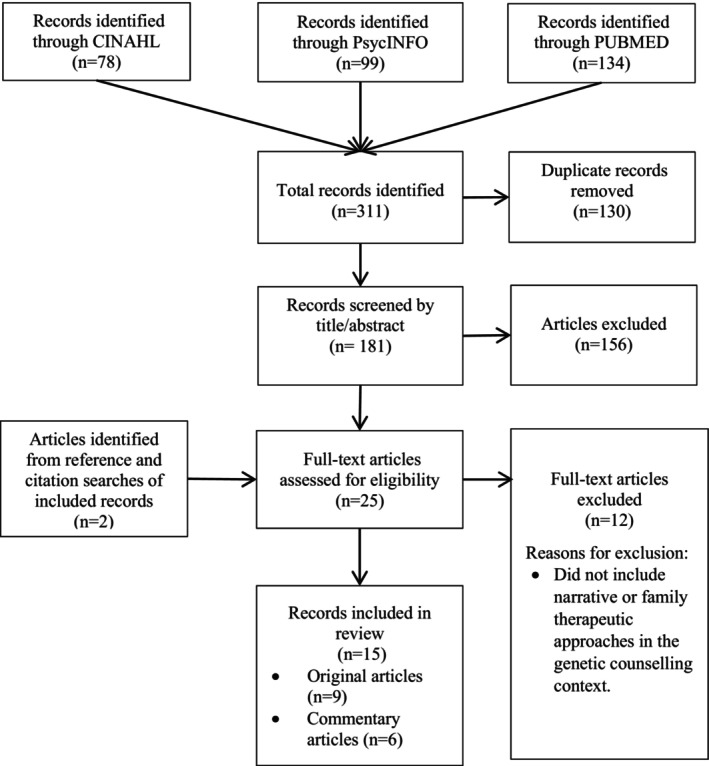
PRISMA flowchart of database and citation search.

Search terms were developed by author A.D. The searches were then conducted by authors J.B. and A.D. and results were imported into Covidence. All articles were screened by authors A.D, A.M.L., and J.B. There were no conflicts regarding inclusion and exclusion of articles between the three authors. Figure 1 outlines the search strategy.

Data from all articles were extracted into Microsoft Word tables. Data extracted from original research articles included study aims, participants, study design, intervention, and major themes/results (see Table S2). Data extracted from commentary articles included a summary of the therapeutic approach and major points made by the author (see Table S3). Data synthesis was performed iteratively by authors A.D. and J.B. from summary tables and returning to full articles.

### 2.4 Quality assessment

Articles were evaluated using the QualSyst tool (Kmet et al., 2004) All articles were reviewed and scored by author J.B. Twenty percent of articles were independently reviewed by author C.W. and any differences were discussed and resolved. No studies were excluded following quality assessment.

## 3 RESULTS

Search terms returned 311 records. After removing duplications (*n* = 130), the remaining articles were screened by title and abstract (*n* = 181), of which 25 underwent full text review. Two eligible articles were identified from reference and citation lists. Fifteen articles met the study inclusion criteria (*n* = 9 original research articles and *n* = 6 commentary pieces). Quality scores ranged from 75% to 95% (Table S4). Study aims, participants, study design, intervention, and major themes/results were summarized for each original research article, and for commentaries the therapeutic approach and practice points made by the author were detailed.

### 3.1 Original research studies

Table 1 provides a summary of original research articles. Studies were conducted in USA (*n* = 4), UK (*n* = 3), and Portugal (*n* = 2). All nine studies assessed the implementation of a structured program or tool that was informed by narrative (*n* = 3) or family therapy (*n* = 6) applied to the genetic counseling context. Four studies assessed the impact of a family therapy interventional tool, that is, the genogram or colored eco‐genetic relationship map (CEGRM) within the standard genetic counseling context (Daly et al., 1999; Peters et al., 2004, 2006, 2012) and two studies evaluated a multifamily discussion group (MFDG) (Mendes et al., 2010, 2015). The three remaining studies examined the feasibility of a narrative therapy group (MacLeod et al., 2018; Spiers et al., 2020; Stopford et al., 2020).

**TABLE 1:** Summary of original research articles relevant to application of narrative and family therapies to genetic counseling practice.

	Study	Purpose and participants	Methodology	Intervention	Major themes/outcomes
Family therapy	Peters et al. (2004)^a^	Utility of CEGRM for family dynamics and social connection. Unaffected women from *BRCA1/2* families; *n* = 20	Cross‐sectional exploratory study. Mixed methods	Semi‐structured interview to construct CEGRM using pre‐drawn pedigree facilitated by research genetic counselor	CEGRM increased empathic connection; promoted insight, awareness, and integration; elicited rich social narratives; opportunity for therapeutic intervention
	Peters et al. (2006)^a^	Utility of CEGRM building on Peters et al. (2004) with larger sample; *n* = 150	Cross‐sectional study. Mixed methods	As per Peters et al. (2004) + religious interaction domains. CEGRM construction facilitated by research GC	CEGRM facilitated insight and revealed previously unrecognized support; promoted emotional expression facilitating insight; opportunity for therapeutic intervention
	Peters et al. (2012)^a^	Utility of CEGRM in a clinical research population of men from testicular cancer families; *n* = 49	Cross‐sectional exploratory study. Mixed methods	As per Peters et al. (2006). CEGRM construction facilitated by research GC	CEGRM useful in understanding social exchanges, health communication roles and men's experiences with a FH of testicular cancer. Identified new potential sources of support for men
	Mendes et al. (2010)^c^	Evaluate use of MFDG in cancer GC. *BRCA1/2* PV carriers; *n* = 9	Exploratory study. Qualitative. Interviews one month post final session	MFDG: four 90‐120‐minute semi‐structured sessions facilitated by trained family therapists	Structure and content were positively evaluated; MFDG format facilitated sharing, bonding and learning different coping strategies
	Mendes et al. (2015)^c^	Utility and feasibility of MFDG in Lynch syndrome or FAP PV carriers; *n* = 19	Non‐experimental exploratory study. Qualitative. Interviews one month post final session	MFDG: four 90‐120‐minute semi‐structured sessions facilitated by trained family therapists	MFDG format promotes familial and non‐familial bonding, a sense of control over health; facilitators' warmth beneficial
	Daly et al. (1999)^a^	Evaluate the genogram to complement GC in females with FH of breast/ovarian cancer; *n* = 38	Pilot study. Quantitative. Standardized genogram information compared to social adjustment scale self‐report scale	Genogram construction to gain insight into family functioning. Genogram interviews conducted by genetic counseling student, nurse, social worker, or health educator	Genogram construction revealed cancer communication patterns and risk perception, family beliefs, support networks. Percentage of close family relationships correlated with social adjustment
Narrative therapy	MacLeod et al. (2018)^b^	Feasibility of a narrative therapy group to complement existing post‐test GC. *n* = 9, post‐HD test result	Mixed methods. PHQ‐9 GAD‐7 assessments pre‐ and post‐session. Qualitative analysis of written comments post‐session	Narrative therapy group including ‘The Tree of Life’ exercise. Co‐facilitated by a clinical psychologist and genetic counselor	Generalized distress decreased pre‐ to post‐session; group shaped a new collective narrative. Major themes: safe space, sense of community, positive coping
	Spiers et al. (2020)^b^	Evaluate participants' experience and feasibility of a GC narrative therapy group. *n* = 12, HD PV carriers	Observational study. Qualitative. Telephone interviews following session	Narrative therapy group with adapted “Tree of Life” exercise. Co‐facilitated by a clinical psychologist and genetic counselor	Major themes: peer support, bonding over shared experiences; benefit of Tree of Life exercise, improved mood, and confidence to share HD status
	Stopford et al. (2020)^b^	Evaluate experience and feasibility of a GC narrative therapy group. *n* = 8, 6 HD PV carriers and two partners	Observational study. Mixed methods. PHQ‐9 and GAD‐7 assessments pre‐ and post‐session. Written feedback post‐session and semi‐structured telephone interview 6–8 weeks post‐session	Narrative group. Adapted “Tree of Life” exercise. Co‐facilitated by a clinical psychologist and genetic counselor	Low distress pre‐ and post‐session. Major themes: safe space facilitated new perspectives; group boosted confidence, optimism and ability to connect due to shared experience

Abbreviations: CEGRM, Colored Eco‐Genetic Relationship Map; FH, family history; GAD‐7, Generalized Anxiety Disorder‐7 scale; GC, genetic counseling; MFDG, multifamily discussion group; PHQ‐9, Patient Health Questionnaire‐9; PV pathogenic variant; SASSR, Social Adjustment Scale Self‐Report.

^a^ USA.

^b^ UK.

^c^ Portugal.

Interventions were most commonly applied in the hereditary cancer setting (*n* = 6) (Daly et al., 1999; Mendes et al., 2010, 2015; Peters et al., 2004, 2006, 2012), or with individuals post‐Huntington disease genetic test result (*n* = 3) (MacLeod et al., 2018; Spiers et al., 2020; Stopford et al., 2020).

Of the nine studies identified, eight assessed the intervention (MacLeod et al., 2018; Mendes et al., 2010, 2015; Peters et al., 2004, 2006, 2012; Spiers et al., 2020; Stopford et al., 2020) using either qualitative (*n* = 3), or mixed‐method approach (*n* = 5). Qualitative assessment included thematic analysis of focus groups, interviews or clinic notes. Interventions were universally acceptable to clients with positive outcomes identified in all eight studies. Key findings included improving insights, facilitating support, enhancing communication, and promoting optimism and coping (MacLeod et al., 2018; Mendes et al., 2010, 2015; Peters et al., 2004, 2006, 2012; Spiers et al., 2020; Stopford et al., 2020).

One study used a quantitative approach in which a questionnaire was developed to assess health beliefs, coping strategies, family communication patterns, and family roles and data were compared to findings from the validated social adjustment scale self‐report (SASSR) scale (Daly et al., 1999).

### 3.2 Group interventions

The five studies evaluating a therapy‐informed group intervention identified safety as a common theme. This intervention facilitated positive outcomes, including bonding through shared experiences, inspiration, and positive adaptations through learning of others' coping strategies (MacLeod et al., 2018; Mendes et al., 2010; Spiers et al., 2020; Stopford et al., 2020).

Participants in four of the five studies found the structure of the group sessions helpful (Mendes et al., 2010, 2015; Spiers et al., 2020; Stopford et al., 2020). The two studies utilizing the MFDG emphasized the importance of striking a balance between structure to gain information, which facilitated clients' sense of control over their health, with open discussions to highlight shared experiences (Mendes et al., 2010, 2015). The MFDG was seen as a useful environment for participants to disclose difficult feelings and diffuse associated tension (Mendes et al., 2015). For example, one participant described how the narrative approach supported him to reflect on the challenges he faced when discussing his negative predictive HD result with his affected brother (MacLeod et al., 2018).

Suggested improvements included allocating additional time for open discussion (Mendes et al., 2015) and incorporating an activity to express feelings of anger and despair (Mendes et al., 2010).

The three studies evaluating the narrative therapy group focused on participants who had undergone predictive testing for Huntington disease. Benefits included the opportunity to shape a new adaptive narrative centered around one's strengths and gifts, and to discuss challenging life experiences (MacLeod et al., 2018; Spiers et al., 2020; Stopford et al., 2020). These three studies used qualitative methods to identify common themes from interviews or written feedback, and showed that the narrative therapy group had a liberating impact on most individuals by fostering connection with others and focusing on their strengths and capacity (MacLeod et al., 2018; Spiers et al., 2020; Stopford et al., 2020). In one of these studies, two of the 12 participants found the group session to be less beneficial as it had been 20 years since they received their test results. However, they acknowledged the potential benefits if they had attended the group session closer to result disclosure (Spiers et al., 2020). The other two studies also used validated scales to assess mood before and after the narrative therapy group session (MacLeod et al., 2018; Stopford et al., 2020). Pre‐ and post‐session levels of generalized anxiety and depression were low with improvements in mood post‐session found in one study, though statistical analysis was not performed due to small numbers (MacLeod et al., 2018).

Overall, the MFDG and narrative therapy groups were well received and participants in the narrative therapy groups were receptive to attending future sessions (MacLeod et al., 2018; Mendes et al., 2010, 2015; Spiers et al., 2020; Stopford et al., 2020). Participants also felt that narrative therapy groups would be applicable to other cohorts (MacLeod et al., 2018; Stopford et al., 2020).

### 3.3 Genogram and CEGRM

Four studies reported that constructing the genogram or CEGRM revealed meaningful information about family functioning, dynamics, and social support (Daly et al., 1999; Peters et al., 2004, 2006, 2012). Three used qualitative methods to identify themes from notes taken during CEGRM construction (Peters et al., 2004, 2006, 2012) with the fourth study employing a questionnaire to develop a genogram, which they compared with results from the validated SASSR scale. Additional open‐ended questions elucidated further detail about family dynamics and psychosocial issues (Daly et al., 1999). In this study, the genogram facilitated discussions about the openness of family communication, family beliefs about familial cancer, and perception of cancer risk (Daly et al., 1999). The validity of the genogram's ability to assess interpersonal relationships was supported by its correlation with the SASSR scale which measures social adjustment by assessing interpersonal relationships within and external to the family (Daly et al., 1999; Weissman & Bothwell, 1976). The genogram was documented to take 30–60 min to complete.

All participants who were invited to complete the CEGRM reported that it was easy to understand and use (Peters et al., 2004, 2006, 2012). The CEGRM elicited rich narratives from the majority of participants, although some individuals found it difficult if they self‐identified as valuing privacy, or struggled to discuss emotive subjects (Peters et al., 2004, 2006). Analysis of open‐ended responses during genogram construction suggested that the process facilitated empathic connection and emotional expressiveness. This prompted personal insight and awareness of family functioning and provided opportunities for therapeutic intervention. Overall, the construction of the CEGRM readily identified individuals' sources of support and barriers to communication (Peters et al., 2006).

It took between 13 and 70 min (average 30 min) to construct the CEGRM with an already completed pedigree. Conclusions regarding the applicability of the CEGRM to standard genetic counseling consultations varied due to the additional time required (Peters et al., 2004, 2006).

### 3.4 Commentary articles

Six commentary articles mentioned psychotherapeutic approaches; one pertaining to narrative therapy (Werner‐Lin & Gardner, 2009), and five to family therapy (Diekmann‐Tapon, 1999; Eunpu, 1997; Kim, 1999; Resta, 1999; Tuttle, 1998). These are summarized in Table 2. Five articles described specific therapy‐informed approaches which can be applied to standard genetic counseling consultations (Diekmann‐Tapon, 1999; Eunpu, 1997; Kim, 1999; Tuttle, 1998; Werner‐Lin & Gardner, 2009) and one of these illustrated the potential use of specific psychotherapeutic activities with the family therapy approach (Tuttle, 1998).

**TABLE 2:** Summary of commentary articles relevant to application of narrative and family therapies to genetic counseling practice.

	Article	Therapeutic approach	Major points
Family therapy	Eunpu (1997)	Family systems based psychotherapeutic techniques in GC to enhance decision‐making and family adjustment. Emphasizes the Intersystem Model, which considers the individual, couple and family system	Client‐centered approach is not sufficient GCs need to be knowledgeable and skilled in various techniques and theoretical approaches as no one theory encompasses all GC scenarios Lack of research assessing psychotherapeutic techniques in different genetic counseling scenarios
	Tuttle (1998)	Experiential family therapy focusing on clients' in‐session experiences and facilitating family growth and change. Goals to increase awareness and expression of feelings. Families motivated to resolve issues to reduce increased anxiety engendered by discussion	Activities promote change as clients see, not just hear, other's feelings Experiential techniques lower defenses and draw upon nonverbal senses to explore difficult issues and foster insight Acknowledges short‐term interventions may be limited and referral for long‐term counseling may be appropriate
	Diekmann‐Tapon (1999)	Object relations family therapy: how individuals relate to each other is based on past relationships and experiences. Aims for family to mature and grow	Prompts the GC to consider the influence of past relationships and experiences on client's behavior and thinking Facilitates self‐awareness and aids decision‐making
	Kim (1999)	Family systems theory, specifically the social ecology model: interaction between a family and its environment. The model assumes the family is influenced by four layers of social subsystems	Knowledge of culture important due to significant influence on family Awareness of the four system layers allows assessment and tailored interventions Approach can organize GC's approach and help anticipate issues Counseling strategies and techniques are needed to complement model
	Resta (1999)	Critiques the social ecology model and examines its limitations in relation to case presented by Kim (1999)	Social Ecology Model formalizes what GCs do GCs cautioned not to stereotype cultural beliefs based on ethnicity Focus on family functioning may cause neglect of individual issues Social ecology model has value, but GC must adapt approach to the individual client or family
Narrative therapy	Werner‐Lin and Gardner (2009)	Narrative therapy: the re‐authoring of an adaptive and empowering personal narrative through therapy and genetic testing of a research participant, “Judith”	“Judith's” narrative constructed in the context of maternal FH of cancer and dying young Identification of a *BRCA1* PV facilitated an alternative narrative of choice and control Case illustrated the power of new adaptive narratives to provide new meaning, direction, greater insight, and improved mental health

Abbreviations: FH, family history; GC, genetic counseling/counselor; PV, pathogenic variant.

Four different family therapy approaches were discussed, including experiential family therapy, the intersystem model, object relations family therapy, and the social ecology model (Diekmann‐Tapon, 1999; Eunpu, 1997; Kim, 1999; Resta, 1999; Tuttle, 1998). The articles all discussed an illustrative genetic counseling scenario based on either a real or hypothetical family. The remaining article discussed the potential use of narrative therapy to facilitate understanding and acceptance of the family medical history alongside integration of genetic test results. The article centered on a female carrier of a pathogenic *BRCA* variant who had undergone long‐term psychotherapy. She had consequently re‐authored an adaptive and empowering personal narrative which incorporated insights from genetic testing results. This case illustrates the power of reauthoring a narrative to provide new meaning and direction in life (Werner‐Lin & Gardner, 2009).

In terms of implementing the psychotherapeutic approach, one article emphasized the importance of maintaining a warm, non‐judgmental presence while focusing on how clients' past relationships and experiences can shape their behavior and thinking, and can generate insight to aid decision‐making (Diekmann‐Tapon, 1999). Some articles suggested that the value of therapeutic approaches lies in helping the genetic counselor organize their thoughts, guide questions and interventions, or anticipate potential issues (Eunpu, 1997; Kim, 1999). Similarly, three articles viewed the therapeutic approach as a lens to understand clients' behavior (Diekmann‐Tapon, 1999; Eunpu, 1997; Kim, 1999). Werner‐Lin & Gardner suggest that by understanding and supporting the development of empowering narratives, genetic counselors can help clients to achieve insight and improve mental health (Werner‐Lin & Gardner, 2009). Other commentaries focused on specific techniques that facilitate positive change and new perspectives (Eunpu, 1997; Tuttle, 1998). Specifically, Tuttle (1998) posited that active techniques (e.g., family drawing, continuums) are an innovative way to gain insight into difficult issues, especially when verbal communication has been ineffective.

## 4 DISCUSSION

This scoping review aimed to describe the applications of narrative therapy and family therapy in genetic counseling, and to document the actual, and perceived value of these approaches in a genetic counseling setting. We found that there is limited literature evaluating the impact of these interventions on client outcomes. Most original studies used qualitative or mixed method approaches to evaluate the use of narrative and family therapy in group or individual genetic counseling settings and were limited to Huntington disease and familial cancer contexts. The interventions were acceptable to clients and associated with positive outcomes including facilitating communication, support, and coping. Benefits identified from the group therapy studies included bonding through shared experiences and being inspired by and learning from the coping strategies of others. The remaining studies utilized tools such as genograms, which revealed meaningful insights into family functioning and support. Articles on interventions using either group therapy or specific tools commented on the time commitment and the practical implications in a clinic setting. Commentary articles discussed the relevance of these methods to the field and proposed specific, hypothetical methods and techniques for applying these theories in practice.

Review of the included studies showed that participants positively evaluated the therapeutically informed group interventions and expressed that the group setting and structure facilitated bonding and support (MacLeod et al., 2018; Mendes et al., 2010, 2015; Spiers et al., 2020; Stopford et al., 2020). Similar findings have been reported among patients with gynecological cancer who participated in an education and group counseling (Sekse et al., 2014). Narrative therapy group participants expressed feelings of empowerment in shaping new narratives (MacLeod et al., 2018; Spiers et al., 2020; Stopford et al., 2020), consistent with studies in other conditions, for example, schizophrenia (Zhou et al., 2023). Narrative therapy has been posited to foster resilience in challenging settings such as following sexual violence (Gomez et al., 2020) and indeed, in the reviewed studies, participants reported appreciating the focus on resilience, strength, and capacity which align well with genetic counseling goals and objectives (Veach et al., 2007). However, it was notable that group interventions in all studies were facilitated by trained family therapists or clinical psychologists, highlighting the need for specialist training. Most genetic counseling training programs are required to teach psychosocial and counseling theory and skills (Accreditation Council for Genetic Counseling, 2024; Human Genetics Society of Australasia, 2023) but genetic counselors may feel underprepared to offer these interventions in practice (Austin et al., 2014; Hartmann et al., 2015; Kessler, 1997). A UK study offered training in facilitating a MFDG to qualified genetic counselors. The training was found to be feasible with genetic counselors quickly developing confidence in family therapy approaches, however only three genetic counselors participated in the program and patient outcomes were not evaluated (Eisler et al., 2017). Additionally, the authors noted that implementation into routine practice would require organizational change including longer or more frequent appointment times and incorporating specialized psychotherapeutic training techniques into professional development (Eisler et al., 2017). The utilization of clinical psychologists in professional supervision for genetic counselors (Kennedy, 2000) may also facilitate adoption of psychotherapeutic approaches in practice.

The construction of a genogram or CEGRM (informed by family therapy) promoted insight and greater awareness of participants' family dynamics (Peters et al., 2004, 2006, 2012) and provided an opportunity for therapeutic intervention (Peters et al., 2004, 2006). Additionally, the CEGRM encouraged the expression of emotions, revealing under‐appreciated emotional ties and supports in men (Peters et al., 2012). As men tend to favor more instructional or problem‐focused coping strategies (Matud, 2004), this tool could be used to foster greater utilization of emotional supports. Furthermore, in some genetic counseling settings a reluctance to discuss sensitive topics and a reliance on information provision has been identified (Hodgson et al., 2010) so the use of such tools may facilitate greater attention to psychological issues.

While effective in promoting personal insight and adaptation to genetic risk, utilization of a genogram or similar tool required an average of 30 min to complete, which multiple studies noted may limit their application in practice (Daly et al., 1999; Peters et al., 2004, 2006, 2012). Family therapy and narrative therapy interventions offered in the group setting involved an additional time commitment of up to 2‐3 hours for single sessions (MacLeod et al., 2018) or may require several sessions (Mendes et al., 2010, 2015), also limiting their utilization in busy clinical settings. Elements of narrative therapy, such as deconstruction and externalization of problems (White, 1993) and family therapy techniques including genograms and role plays (Asen & Scholz, 2010) could be applied within a standard genetic counseling session; however, there is a lack of literature evaluating feasibility, how it might translate in practice and the subsequent impact on clients.

The commentary articles commonly emphasized the value of narrative and family therapeutic approaches in promoting positive change, fostering an appreciation for different perspectives, and providing innovative ways of exploring challenges (Diekmann‐Tapon, 1999; Eunpu, 1997; Tuttle, 1998; Werner‐Lin & Gardner, 2009). It is also argued that a therapeutic lens can provide structure in organizing thoughts, guiding questions, and anticipating potential issues for a client or family (Diekmann‐Tapon, 1999; Eunpu, 1997; Kim, 1999), though Resta (1999) cautioned against the over‐reliance on a single therapeutic lens due to its potential to lead to stereotyping or making assumptions (Resta, 1999). Eunpu (1997) further states that a client‐centered approach is not sufficient to achieve optimal outcomes for clients and that genetic counselors need to be knowledgeable and skilled in various techniques and theoretical approaches. She calls out the lack of research assessing the psychotherapeutic techniques that work best in different genetic counseling scenarios and emphasizes the critical need for this research, a sentiment also noted in earlier pieces (Diekmann‐Tapon, 1999; Eunpu, 1997; Kim, 1999). It is important to note that such interventions are unlikely to be embraced by the entire profession given historic hesitancy to acknowledge the psychological aspect of the role (Eunpu, 1997). Nonetheless, genetic counseling helps “people understand and adapt to the medical, psychological, and familial implications of genetic contributions to disease” (Resta et al., 2006), and thus it is crucial that we find time for interventions which meet this goal.

Strengths of this study include an extensive literature search in all relevant databases, and the qualitative assessment of each study. Limitations of this study include the existence of multiple terms and techniques which fall under the umbrella of family or narrative therapy interventions and thus some papers may have been missed. The inclusion of commentaries, as opposed to focusing solely on original research, introduces an element of potential bias. Finally, the finite number of identified papers limits the study findings and the ability to draw conclusions.

## 5 PRACTICE IMPLICATIONS AND FUTURE RESEARCH DIRECTIONS

This review describes the value of psychotherapeutic interventions in addressing complex relationships and communication patterns inherent in adapting to genetic information and supports previous studies which describe the value of psychotherapeutic interventions in genetic counseling (Austin et al., 2014; Kessler, 1979; Veach et al., 2007). However, we identify the significant gaps in the literature regarding the specific impacts of psychotherapeutic approaches on genetic counseling outcomes in clinical consultation settings. Future research to address these gaps could utilize standardized outcome scales specific to genetic counseling to evaluate such techniques, which could include perceived personal control scale (Berkenstadt et al., 1999), the genetic counseling outcomes scale (McAllister et al., 2011), and genetic counseling satisfaction scale (DeMarco et al., 2004). Other psychological and social outcome measures could also be applied (Kasparian et al., 2007; Resta, 2019).

To substantiate the value of psychotherapeutic methods in genetic counseling, future studies should focus on evaluating the impact of such methods on genetic counseling outcomes in the setting of standard consultations. Evidence that psychotherapeutic approaches, such as family and narrative therapy, are effective in this setting is critical to informing best practice in standard genetic counseling sessions across all subspecialties. Ideally, randomized controlled trials are needed to determine this, however, other quantitative and qualitative studies would be valuable (Kasparian et al., 2007).

Integrating psychotherapeutic group interventions into publicly funded clinical genetics departments aligns with the goals of genetic counseling and is likely to improve family communication, family dynamics, and help patients to reframe their narratives around their condition and/or their risk. However, there may be logistical challenges given the time commitment.

In conclusion, there is limited research describing the application of and assessing the value of both family and narrative therapy in the genetic counseling context. Original research demonstrated that both approaches are acceptable to clients, and group therapy can facilitate disclosure of challenges, identification of shared experiences, nurture mutual support and provide inspiration to promote health coping. Both therapies offer the client the opportunity to distance themselves from their current problems to gain a new perspective, thereby increasing empowerment and self‐efficacy. Narrative therapy specifically, is focused on understanding the multiple stories which shape an individual and the generation of alternative stories which may provide more satisfying or meaningful ways to understand their condition (Denborough, 2012). Family therapy has the potential to reveal aberrant communication patterns and barriers which can promote insight and positively impact communication (Carr, 2016). Given the alignment between these approaches and the overall goals of genetic counseling, additional research is needed to evaluate their impact on client outcomes and further demonstrate their potential application in the context of standard genetic counseling sessions.

## AUTHOR CONTRIBUTIONS

Aimee Dane and Jennifer Berkman were involved in study design, data extraction, and analysis and manuscript writing. Emily DeBortoli and Courtney K. Wallingford contributed to data analysis and quality assessment. Tatiane Yanes and Aideen McInerney‐Leo contributed to manuscript writing and editing. All authors critically edited the manuscript.

## CONFLICT OF INTEREST STATEMENT

Aimee Dane, Jennifer Berkman, Emily DeBortoli, Courtney Wallingford, Tatiane Yanes, and Aideen McInerney‐Leo declare that they have no conflict of interest.

## ETHICS STATEMENT

Human studies and informed consent: Ethical approval was not required as only publicly available literature was used. No experiments were performed on human or animal subjects.

Animal studies: No animal studies were carried out by the authors for this article.

## Supporting information


Appendix S1

Table S1

Table S2

Table S3

Table S4

## Data Availability

All data and materials are available from the corresponding author (J.B.) upon request.
