# Supplementary material for: Narrative therapy and family therapy in genetic counseling: A scoping review
Source: J Genet Couns. 2024 Jun 20;34(2):e1938. doi: 10.1002/jgc4.1938 (PMC11907181; doi:10.1002/jgc4.1938)
Supplement: Supplementary file 2 — Table S1 [file JGC4-34-0-s005.docx]

**Table S1**: *Search terms used to identify articles which may be suitable for inclusion in a review of narrative and family therapies in genetic counseling*

| **Most recent search date** | **Database** | **Search terms** |
| --- | --- | --- |
| 12.4.24 | CINAHL | AB genetic counsel* AND AB narrative |
| 12.4.24 | PsycINFO | AB genetic counsel* AND AB narrative |
| 12.4.24 | PubMed | ("genetic counsel*"[Title/Abstract]) AND ("narrative"[Title/Abstract]) |
| 12.4.24 | CINAHL | AB genetic counsel* AND AB (family therapy OR family theory OR family system*) |
| 12.4.24 | PsycINFO | AB genetic counsel* AND AB (family therapy OR family theory OR family system*) |
| 12.4.24 | PubMed | (genetic counsel*[Title/Abstract]) AND (family therapy[Title/Abstract] OR family theory[Title/Abstract] OR family system*[Title/Abstract]) |
| 12.4.24 | CINAHL | colored eco-genetic relationship map OR CEGRM |
| 12.4.24 | PsycINFO | colored eco-genetic relationship map OR CEGRM |
| 12.4.24 | PubMed | colored eco-genetic relationship map OR CEGRM |
