# Supplementary material for: Narrative therapy and family therapy in genetic counseling: A scoping review
Source: J Genet Couns. 2024 Jun 20;34(2):e1938. doi: 10.1002/jgc4.1938 (PMC11907181; doi:10.1002/jgc4.1938)
Supplement: Supplementary file 3 — Table S2 [file JGC4-34-0-s001.docx]

**Table S2:** *Summary of original* *articles relevant to application of narrative and family therapies to genetic counseling practice*

Original research articles

| **Study** | **Purpose & Participants** | **Study Design** | **Intervention** | **Major Themes/Results** | **Strengths and Limitations of Study** |
| --- | --- | --- | --- | --- | --- |
| **Peters et al. 2004** | An exploratory study to determine the compliance, feasibility and utility of using the Colored Eco-Genetic Relationship Map (CEGRM). The CEGRM is a tool designed to understand family dynamics and social connection.  Unaffected women from a family with a known *BRCA1* or *BRCA2* deleterious mutation and a first or second degree relative diagnosed with a *BRCA*-associated cancer.  n = 20. Mean age 44 years (29 – 56 years). United States of America | Exploratory cross-sectional study. Mixed methods. Mainly quantitative with some qualitative measures and analysis.  Qualitative data was analysed using an iterative process to identify patterns, processes and relationships in the data. | CEGRM was constructed using participants previously collected genetic pedigree. Researcher used a semi- structured interview to construct the CEGRM by placing stickers at appropriate locations to illustrate who participant a) shares information about cancer/genetics, b) shares feelings about cancer risk, c) receives material/physical support (mutuality in these interactions was also assessed). Individuals who were seen as information disseminators or information blockers were also indicated with stickers. The researcher noted down verbal comments made during CEGRM construction (i.e. narratives, experiences, feedback on the process of CEGRM construction) | 1. CEGRM usually took less than 30 mins to complete (range = 13 – 50 mins) with a previously completed pedigree.  2. Compliance – all 20 women who were invited completed the CEGRM  3. CEGRM was perceived as easy to understand  4. CEGRM was perceived as comfortable to construct  5. Most participants found it easy to place stickers to construct CEGRM  6. CEGRM seemed to facilitate easeful discussion about family relationships. Most participants rated ‘ease of talking’ to be high  7. Usefulness of CEGRM in eliciting family narratives was varied. Those who felt it was less useful described themselves as ‘private.’  8. All participants perceived that they talked more than half the time of the CEGRM construction  9. Authors concluded the following about the CEGRM based on verbal comments:  - increased empathic connection with the researcher  - promoted insight, awareness and integration  - elicited rich social narratives  - highlighted the importance of mutuality in relationships  - is useful in understanding family and non-family social connections  - provided the opportunity for therapeutic intervention when difficult feelings arose | Strengths:  - Mixed quantitative and qualitative methods which provides richer data and is grounded in participants’ experience  Limitations:  - Small, biased sample – participants mostly Caucasian, highly-educated and motivated. Limited generalisability.  - Potential ‘halo effect’- evaluation of CEGRM was conducted by same individuals providing clinical genetics service. |
| **Peters et al. (2006)** | An exploratory study to determine the compliance, feasibility and utility of the CEGRM by building on Peters et al. (2004) and extending to a larger sample of women with hereditary breast and ovarian cancer.  Unaffected women from a family with a known *BRCA1* or *BRCA2* deleterious mutation.  n = 150 (mean age 39 years, range 22 – 56 years). 20 women from Peters et al (2004) also included in this study. United States of America | Exploratory cross-sectional study. Mixed methods. Mainly quantitative but some qualitative analysis.  Qualitative data was analysed using an iterative process to identify patterns, processes and relationships in the data. | As per Peters et al. (2004) with added domain of religious/spiritual interactions. Further detail was gathered regarding information interactions (i.e., people who tended to gather information, disseminate information, and block information). | 1. CEGRM took 13 – 60 mins to complete (mean = 30 mins)  2. 100% compliance  3. Most participants found it easy to understand and use, and found it comfortable to construct  4. Majority of participants perceived they talk more than half the time  5. Perceived ease in taking about relationships was high, but some participants appeared to find it difficult because the topic was upsetting or the participant self-identified as being a private personality  6. The CEGRM readily identified relationships and sources of support  7. Authors concluded that CEGRM:  - facilitated insight and revealed previously unrecognised sources of support - supported participants to feel they were the expert of their lives - promoted emotional expressiveness which in turn facilitated insight and an opportunity for therapeutic intervention  8. Authors conclude that the CEGRM is complementary to standard genetic counselling | Strengths:  - Large sample size which means that results are more robust.  - Results were in line with Peters et al., (2004) which adds weight to findings.  - Mixed quantitative and qualitative methods which provides richer data and is grounded in participants’ experience  -  Limitations:  - Potential ‘halo effect’- evaluation of CEGRM was conducted by same individuals providing clinical genetics service.  - Feedback from some participants suggested researchers’ warm demeanour may have encouraged conversation and expressiveness rather than the CEGRM itself. Study unable to disentangle true contribution of CEGRM to study outcomes. |
| **Peters et al. (2012)** | To determine the acceptability, feasibility and utility of CEGRM in a clinical research population of men^1^  Eligibility criteria were men from a family with two or more confirmed cases of testicular cancer. Affected and unaffected men from such families were eligible to participate.  n = 49 from nineteen families. Age 16 – 79 years, mean 40 years. United States of America | Exploratory, cross-sectional study including quantitative and qualitative measures^2^ to evaluate compliance, feasibility and utility of CEGRM.  Qualitative data was analysed using an iterative process to identify patterns, processes and relationships in the data. | As per Peters et al. (2006) | 1. Compliance was high – 100% completion of CEGRM  3. Understanding and comfort were perceived to be high  4. Stickers were generally perceived as easy to use though some difficulty with treatment related neuropathy  5. Ease of talking was rated as high, as was the CEGRM’s encouragement of family narratives 6. Most participants felt they talked more than half of the time.  7. Authors concluded that the CEGRM was useful in understanding social exchanges, health communication roles and men’s experiences with a family history of testicular cancer. Some information gained from CEGRM may not have been gained from usual communication.  2. Average time to completion was 40 mins (range was 15- 70 mins) | Strengths:  - Focused on an under studied population (men with family history of testicular cancer).  - Mixed quantitative and qualitative methods which provides richer data and is grounded in participants’ experience  Limitations:  - Potential ‘halo effect’- evaluation of CEGRM was conducted by same individuals providing clinical genetics service. |
| **Mendes et al. (2010)** | To determine the value and suggested changes required for incorporation of a multifamily discussion group (MFDG) into the genetic counselling protocol for hereditary cancers.  Inclusion criteria - families with at least one family member with a deleterious *BRCA1/2* mutation.  Three families. n = 9 (all female). Age 24 – 74 years (mean age 43.5). All  participants had a deleterious *BRCA1* or *BRCA2* mutation. Portugal. | Qualitative. Focus group semi-structured interview conducted one month post last session by program facilitators.  Focus group interviews covered the content and activities of the program, perceived benefits and suggestions for improvement. Interview was video recorded, transcribed and analysed for content. | The MFDG aimed to be a complementary family focused tool to address psychosocial adjustment. MFDG structured as four 90 – 120 minute semi-structured sessions run by facilitators trained and experienced in family therapy. MFDG were run on a Saturday. Sessions covered the impact of familial genetic risk, medical information, resources, embracing family identity, problem-solving techniques, stress management strategies. | 1. Structure and content were positively evaluated – particularly the mix of structured and unstructured components  2. Participants expressed the perceived benefit of MFDG format which facilitated sharing, bonding and learning different coping strategies  3. Information session was a non-confrontational way of accessing accurate information  4. Role of facilitator – encouraging open conversation while re-directing when necessary  5. Suggestions for improvement | Strengths:  - All participants attending MFDG attended the evaluation focus group –  Qualitative methodology allowed  for rich understanding of  participants’ experience  Limitations:  - Facilitator conducted the evaluation which may have biased responses (‘halo effect’)  - Small sample of only females with *BRCA1* or *BRCA2* mutation (rather than having some family members who had not had testing). Limited generalisability  - Biased sample – motivated to attend MFGD |
| **Mendes et al. (2015)** | To determine the value of a multifamily discussion group (MFDG) and its feasibility as a complementary tool to cancer genetic counselling. Families with at least one family member with a deleterious colorectal cancer mutation (Lynch syndrome or familial adenomatous polyposis (FAP))  Four families. Families had between 3-6 members present. n = 19 (13 females, 6 males). Age 14 – 56 years (mean age 33.5). Portugal | Qualitative. Focus group interviews were conducted with all participants one month post last session by the program facilitators. Focus group interviews covered the content and activities of the program, perceived benefits and suggestions for improvement. Thematic analysis by independent researcher and first author (unclear if also facilitator). | The MFDG was run as four semi-structured 90 – 120-minute sessions on a Saturday. Sessions covered the impact of familial genetic risk, medical information, resources, embracing family identity, problem-solving techniques, stress management strategies. | 1. Participants expressed the perceived benefit of MFDG format for familial and non-familial bonding  2. Sense of control over health management from education session  3. Benefit of facilitators’ warm and informal approach | Strengths:  - All participants attending MFDG attended the evaluation focus group  - Qualitative methodology allowed for rich understanding of participants’ experience  - FAP and Lynch syndrome families combined – demonstrates commonality can be found across conditions  Limitations:  - Facilitator conducted the evaluation which may have biased responses  - Small sample with overrepresentation of females – limited generalisability  - Biased sample – motivated to attend MFGD |
| **MacLeod et al. (2018)** | To explore the feasibility of offering a narrative group in the context of the existing predictive follow up genetic counselling clinic for HD. To determine participants’ experience of the program and  whether they would recommend  participation to others.  Individuals who had tested  negative for HD mutation in the  last 5 years through the  Manchester Centre for Genomic  Medicine.  n = 9 (6 men, 3 women). Age 31  – 66 years. United Kingdom | Mixed methods but mainly qualitative. Participants were asked to complete the Patient Health Questionnaire (PHQ-9) and the Generalised Anxiety Disorder (GAD-7) assessments before and immediately after the group session. Written feedback was collected using semi-structured questions. Questions were about the experience of taking part in the group, suggestions for improvement and comments on who they felt would benefit most from this type of follow up appointment. Thematic analysis was used to interpret the free text comments. | Narrative therapy group.  Aims of the narrative group were to foster resilience and strengthen existing coping using an approach drawn from narrative therapy. Included an exercise adapted from narrative therapy - ‘The Tree of Life.’ Narrative therapy group was co-facilitated by a clinical psychologist and genetic counsellor within the National Health Service (NHS) clinical genetics setting | Overall findings:  - Generalised anxiety and depression appeared to decrease from pre- to post-session but statistical analyses were not conducted due to  small sample size  - Participants spoke of feeling isolation, guilt and shame and that the group helped to shape a new collective narrative  Major themes:  1. Safe space of the group  2. Sense of community – bonding over shared experiences  3. Emphasis on positive coping – a way of talking about difficult experiences while being able to focus on resilience.  4. Suggestions for future sessions – participants felt that group has relevance to anyone who has been affected by a family history of HD | Strengths:  - Non-facilitating member of research team conducted evaluation – limits bias of ‘halo effect’  - Mixed quantitative and qualitative methods which provides richer data and is grounded in participants’ experience. Use of PHQ-9 and GAD-7 adds weight to positive impact of group  - Good representation of male participants which is lacking in other studies  Limitations:  - Small sample size – limited generalisability  - Evaluation may have been limited by receiving written feedback only, rather than interviews or focus group |
| **Spiers et al. 2020** | To evaluate the participants’ experience with a genetic counselling narrative group. To determine whether attendance at a single narrative group was perceived as helpful. To determine the feasibility of integrating attendance at a single narrative group into standard genetic counselling practice with a view to maximising psychological adaptation. Individuals with a positive Huntington disease (HD) predictive test result and had taken part in a genetic counselling narrative group between Nov 2017 and Feb 2018.  n = 12 (10 female, 2 male). Age 27 – 59 years). United Kingdom | Qualitative. Telephone interviews following participation in narrative group. Questions were about the narrative group and the impact on the participants’ lives.  Interpretative phenomenological analysis was used to explore participants’ experience. | Narrative group with the aim to help build resilience and find new ways of making sense of their HD status. Group facilitated by a clinical psychologist and genetic counsellor trained in approach.  Adapted tree of life exercise was used to facilitate a discussion about strengths and support systems. | Major themes:  1. Power of the group – peer support, bonding over shared experiences, inspiration by seeing other group members coping/adapting  2. Active elements of the narrative exercise – benefit of the tree of life exercise  3. Subsequent impact of the session – increased mood, freeing impact of session and confidence to disclose HD status  4. Another voice: The experience of two participants who felt the session was less beneficial | Strengths:  - Telephone interviews conducted by independent researcher -reduces bias of ‘halo effect’  - Qualitative design allowed for rich descriptions of participants’ experiences  Limitations:  - Small sample size and high proportion of female participants – limited generalisability  - Biased sample – motivated to attend narrative group |
| **Stopford et al. (2020)** | To evaluate a narrative group lead by clinical psychologist and genetic counsellor trained in approach. To understand the feasibility and value of integrating narrative groups into standard genetic counselling practice. Individuals with a positive HD predictive testing result attending a single narrative group session.  n = 8 (3 female, 3 male, 2 male partners not at-risk of HD). Age 19 – 45 years. United Kingdom | Mixed methods but mainly qualitative. Data collected via written feedback forms following the session and semi-structured telephone interview 6-8 weeks post-session. Questions included participants’ experience and reaction to the session, and general mental wellbeing. Thematic analysis was undertaken by an independent researcher  and the program facilitating genetic counsellor. Generalised Anxiety Disorder 7 (GAD7) and Patient Health Questionnaire 9 (PHQ9) scales were used before the session and 2 weeks post-session. Two participants were unable to partake in the telephone interview. | Narrative group facilitated predominantly by clinical psychologist – known to participants. Genetic counsellor also helped facilitate the session. Session used an adapted ‘Tree of Life’ exercise with uses leaves, roots, branches, etc of a tree as metaphors to represent different aspects of peoples’ lives. Focus of exercise is for participants to broaden how they see themselves and the possibilities for living their life. | Levels of anxiety and depression as per GAD7 and PHQ9 were low pre- and post-session, most scoring in the ‘mild’ range. Pre- to post-session changes were not apparent and therefore statistical analyses were not employed.  Major qualitative themes:  1. Experiences during the session (safe space, benefit of comparison by way of gaining new perspectives, the importance of structure to the group)  2. Post-session reflections (positive evaluation of the narrative group, group was enjoyable, boosted confidence and optimism, ability to connect with others with shared experience)  3. Future recommendations (willingness to attend future groups, applicability to other conditions, suggested improvements) | Strengths:  - Telephone interviews conducted by independent researcher – reduces bias of ‘halo effect’  - Mixed quantitative and  qualitative methods which provides richer data and is grounded in participants’ experience. Use of PHQ-9 and GAD-7 adds weight to positive impact of group  - Even number of male and female participants – greater generalisability  Limitations:  - Small purposively selected sample – limited generalisability - Biased sample – motivated to attend narrative group |
| **Daly et al. (1999)** | To evaluate the use of a genogram as a complement to genetic counselling sessions  with women at increased risk of breast and/or ovarian cancer. To validate the information gained from a genogram by comparing to the Social Adjustment Scale Self-Report (SASSR). SASSR is an instrument for measuring social adjustment. To determine the efficacy of the genogram in assessing family relationships. Women with a family history of breast and/or ovarian cancer attending a multidisciplinary team program that includes:  - education sessions on  medical and genetic information about breast and ovarian cancer  - consultation with genetic counsellor to review family history and offer genetic testing if appropriate  - consultation when results available regarding risks and appropriate management - consultation with social worker regarding psychosocial sequelae  n = 38 (all females). Mean age - 46.6 years. | Quantitative. Genogram interviews conducted by genetic counselling student, nurse, social worker or health educator. | Genogram construction and interpretation was informed by family systems theory. The genogram seeks to provide insight into family functioning and can be used to visually illustrate the transmission of behaviour patterns over generations. The interview questions for constructing the genogram were standardised. | Significant detail about the participant’s relationships and family dynamics was gleaned from constructing the genogram. The genogram facilitated conversations about the degree of open communication about cancer, the family’s beliefs about the implications of cancer in the family, and the influence of having a close relationship with a family member with cancer on their perception of cancer risk. The percentage of *close* or *very close* family relationships was significantly correlated with social adjustment as measured by the SASSR. The authors suggest that this validates the questions used in the genogram and that the genogram is accurately capturing family relationships. The authors argue that by standardising the content and structure of the genogram construction, this can be used more readily in comparative research and counselling settings. The authors point out that the genogram can be used to identify support networks or likely difficulties within the family. Authors suggest that it is an innovative adjunct to genetic counselling practice. | Strengths:  - Standardising the procedure for genogram allows for comparison across studies  Limitations:  - Self referred, highly motivated and educated sample - Potential ‘halo effect’ due to evaluation being conducted by same individuals providing clinical genetics service. |

^1^ This study included other research aims not relevant to the current literature review and therefore have not been described

^2^ This study included other outcome measures not relevant to the current literature review and therefore have not been described
