# Supplementary material for: Narrative therapy and family therapy in genetic counseling: A scoping review
Source: J Genet Couns. 2024 Jun 20;34(2):e1938. doi: 10.1002/jgc4.1938 (PMC11907181; doi:10.1002/jgc4.1938)
Supplement: Supplementary file 4 — Table S3 [file JGC4-34-0-s004.docx]

**Table S3:** *Summary of therapeutic approach and major points of commentary articles relevant to application of narrative and family therapies to genetic counseling practice*

| **Family Therapy** |
| --- |
| **Eunpu (1997)** |
| *Therapeutic approach:*   - Systemically-based (i.e. family systems based) psychotherapeutic techniques for use in genetic counseling that enhance decision-making and facilitate family adjustment to genetic disorders and/or genetic risk. - Particularly emphasises the Intersystem Model, which proposes that the individual, couple and family system all need to be focused on to effect positive change.   *Genetic counseling context:*   - Article focuses on multiple hypothetical genetic counseling scenarios and demonstrates the use of various techniques aimed at addressing the individual, couple and family system.   *Major points:*   - Author argues that given family is the unit of treatment in genetics, the use of systemically based psychotherapeutic techniques are very relevant, especially in families where the family's integrity is considered more important than that of the individual. Acknowledges that for families where individuals' needs are more important, a different approach may be needed. - Author argues that if genetic counselors are going to own the importance of psychosocial support in their roles, some negotiation is likely to be required to free up time and space for this work to be carried out with clients. Author argues that a client-centered approach is not sufficient for genetic counseling and genetic counselors need to be knowledgeable and skilled in various techniques and theoretical approaches. - Discusses the relevance of ecomaps and genograms to (a) understanding family members' interactions with other entities (e.g., extended family, church) and, (b) understanding family dynamics in line with a family therapy approach. - Author acknowledges that no one theory encompasses all genetic counseling scenarios and advocates for genetic counselors having a theoretical basis for their work. Author calls out the lack of research assessing the psychotherapeutic techniques that work best in different genetic counseling scenarios and emphasizes the critical need for this research. |
| **Tuttle (1998)** |
| *Therapeutic approach:*   - Experiential family therapy which focuses on clients' in-session experiences and how this can produce family growth and change. - Goals of this therapy are to increase awareness and expression of feelings, to increase flexibility and spontaneity in how family members respond to problems. - A major focus of this approach is the experiences the client/s have in the session. Several techniques are used to provide such experiences:   - Kvebaek technique - uses a chess-like board and moveable pawns to demonstrate closeness/distance.   - Family drawings/sculptures - to illustrate family dynamics.   - Continuums - using the room or a piece of paper to illustrate where one feels they sit on a given continuum.   - Empty chair technique - for role plays or for less confrontational way of speaking to someone who is or is not present.   - These techniques prompt expression of feeling and is usually coupled with an increase in anxiety. It is said that to try to relieve this anxiety, families are motivated to resolve the presenting issue. |
| *Genetic counseling context:*   - Father who had brought his son, with autism, to a genetics appointment to assess if there is a syndromic cause of autism. Genetic counselor notices the wife's absence and a few comments made by the father that his marriage may be experiencing difficulties. - Article written about a hypothetical second appointment with genetic counselor using experiential family therapeutic approach.   *Major points:*   - Use of family drawing where each family member is depicted by circle can illustrate family dynamics. By comparing drawings and asking about why individual chose to draw circles in given arrangement can foster communication with a different perspective. Further healing can be promoted by asking family to draw circles depicting how they would like their family to function. The process of *seeing* each other's feelings rather than hearing them, may promote change. The discussion of the drawings can increase understanding of each other. The same or more powerful outcome can be promoted by using family sculpture. - Empty-chair technique can allow one or both members of a couple to speak uninterrupted about how they feel. - Role reversal can be used to gain different perspectives. - Continuums can be used to illustrate where each family member sits on a continuum of, for example, sense of control about their child's diagnosis. - Author acknowledges the limits of such short-term interventions and that referral to long-term counseling may be appropriate. - Author suggests that these techniques could apply to cancer counseling and support groups. - Author argues that experiential techniques lower defenses and draw upon nonverbal senses to explore difficult issues in an innovative way that can foster insight. - Author also argues that the techniques are especially useful if verbal communication appears ineffective. - Author argues that techniques allow us to maximize our efficiency in exploring psychosocial issues and that techniques are unlikely to be relevant for every client but that clinical judgement should be used. |
| **Diekmann-Tapon (1999)** |
| Therapeutic approach:   - Object relations family therapy (ORFT) which combines psychodynamic object relations theory with family therapy. - The theory posits that how individuals relate to each other is due to their past relationships and experiences. These past experiences are internalised and are called *objects*. - The theory posits that early childhood experiences in relationship with parents is crucial but can be modified by experiences later in life. - The stance of the ORF therapist is non-directive, and one of safety, warmth and the willingness to non-judgmentally hold anything that is brought up by the family. - The aim of the therapy is to allow the family to mature and grow. ORFT posits that repressed longing for a secure attachment to a parent can be repressed and result in a 'false self'   e.g. an individual who states that they do not need to be loved, when unconsciously this is what they are longing for due to past negative relationships.  *Genetic counseling context:*   - Couple with recurrent trisomy pregnancies. First pregnancy had trisomy 18 and couple had termination of pregnancy. Couple were seen in clinic with another pregnancy that later was determined to be at increased risk for trisomy 13. - Article written as suggested additions to genetic counseling session by using ORF therapeutic approach   *Major points:*   - Author argues that the therapy's focus on past relationships would have triggered the genetic counselor to explore past experiences to a greater degree e.g., couple's experience of first pregnancy and termination. - Author argues that by identifying an apparent lack of anxiety in the husband, the genetic counselor could have explored the husband's emotions to allow him to become more in touch with his feelings, which may in turn have had a positive effect on the couple's relationship. - By creating a safe, warm, nonjudgmental space, the genetic counselor could have allowed the couple to voice their fears and express their feelings to a greater degree. - Author argues that approach is relevant to genetic counseling because it is non-directive. - Author argues that ORFT's focus on transference and countertransference would have prompted the genetic counselor to examine the feelings evoked in herself/himself by the couple, and to use these feelings to better the therapeutic alliance. - Author acknowledges criticism that a psychoanalytic approach is not applicable to genetic counseling because it is generally a long-term approach. Author argues that the case study demonstrates its applicability because it would have prompted the genetic counselor to consider the influence of past relationships and experiences on client's behavior and thinking, which can facilitate self-awareness and aid decision-making. |
| **Kim (1999)** |
| *Therapeutic approach:*  Family systems theory, specifically the Social Ecology Model which looks at the interaction between a family and its environment.   - The model assumes that the family is influenced by larger social subsystems (e.g. culture, healthcare system, economy). - The model describes four layers of systems: microsystem (family functioning), mesosystem (groups the family belongs to e.g., church, extended family), exosystem (systems such as school, healthcare system) and macrosystem (large systems such as political, health, cultural, religious).   *Genetic* counseling context:   - An Indian couple who bring their two month old daughter who has been diagnosed with oculocutaneous albinism to the genetics clinic to understand the condition, find out about the familial implications, find a support group and learn about research on albinism. - Article is written about a hypothetical second appointment with the couple illustrating how the therapeutic approach could be used. The article acknowledges that such an approach would not have been possible in the first appointment due to the time and physical constraints of the first appointment with the geneticist.   *Major points:*   - Author argues that knowledge of culture is important for genetic counselors to be aware of because it can have a significant influence on the family (i.e. in the form of the exosystem). - Author argues by being aware of the four layers of systems the genetic counselor can assess each one and provide tailored interventions. For example, by enquiring about the mother-father and parent-child interactions, as well as the parent's acceptance of their child's disability, the genetic counselor can tailor the discussion to their needs and determine the need for referral for additional psychological support. By enquiring about the mesosystem, the genetic counselor can assess the family's connectedness with society, social supports from friends or extended family. Again, this could lead to tailored support i.e., assisting the family to effectively communicate their daughter's diagnosis with important others. In enquiring about the influence of the exosystem and macrosystem, the genetic counselor can explore how the family are responding to support or lack of support from their daughter's school, cultural messages about acceptance or workplace support around attending appointments, etc. - The author acknowledges that she raises more questions than she provides answers for but argues that the approach is useful in organizing the genetic counselor's thoughts in working with a family facing a genetic disorder. - The author acknowledges that the approach is not a counseling theory that suggests techniques for working with a family, and instead it helps the genetic counselor to anticipate the various issues a family may be facing. It also highlights the many levels a genetic counselor may influence the family's ability to cope. - The author therefore acknowledges that counseling strategies and techniques are needed in addition to the Social Ecology Model. |
| **Resta (1999)** |
| *Theoretical approach:*   - Critiques the Social Ecology Model and examines its limitations in relation to case presented by Kim (1999)   *Genetic counseling context:*   - A commentary of Kim (1999) paper   *Major points:*   - Author argues that the Social Ecology Model formalizes what genetic counselors already do. - Cautions genetic counselors not to stereotype cultural beliefs based on ethnicity. - Author argues that by focusing on family functioning, the genetic counselor may neglect focusing on the individual. - Argues that the Social Ecology Model has value, but the genetic counselor must have knowledge of a variety of counseling techniques and theories so as to adapt their approach and understanding to the individual client or family. |
| **Narrative Therapy** |
| **Werner-Lin & Gardner (2009)** |
| *Theoretical approach:*   - Narrative therapy.   *Genetic counseling context:*   - Article was written by researchers who interviewed a participant ‘Judith’ as part of a qualitative study about integration of genetic testing results and family medical histories. Judith had been through long-term psychotherapy and through her research interview illustrated how she had re-authored an adaptive and empowering personal narrative through therapy and genetic testing.   *Major points:*   - By viewing ‘Judith’s’ narrative from the context of a multi-generation family, the authors argued that one can see how Judith’s narrative was shaped. Judith’s mother was diagnosed with breast and ovarian cancer and died young. From her family history, Judith believed she would die of cancer young. With the availability of genetic testing, Judith was able to construct an alternative narrative of choice and an element of control by learning she has a *BRCA1* mutation. This alternative narrative empowered Judith to decide to have risk- reducing bilateral salpingo-oopherectomy to reduce her risk of ovarian cancer, thereby increasing the chances of living to see her children have their own children. Judith also begins to construct a new trajectory in partnership with her husband who has a healthy and long-lived family history. - The authors provide comment on the power of facilitating new adaptive narratives in the genetic counseling context by illustrating how it provided Judith with new meaning and direction in life. - Authors argue that the availability of genetic testing allows for the creation of a new and empowering narrative that genetic counselors can support and facilitate, which can in turn lead to insight and greater mental health. |
