# Supplementary material for: Narrative therapy and family therapy in genetic counseling: A scoping review
Source: J Genet Couns. 2024 Jun 20;34(2):e1938. doi: 10.1002/jgc4.1938 (PMC11907181; doi:10.1002/jgc4.1938)
Supplement: Supplementary file 5 — Table S4 [file JGC4-34-0-s002.docx]

**Table S4.** Quality assessment of records, using QualSyst Tool [Kmet et al, 2004]

| **Quantitative** | **1. Question / objective sufficiently described?** | **2. Study design evident and appropriate?** | **3. Method of subject / comparison group selection or source of information / input variables described and appropriate?** | **4. Subject (and comparison group, if applicable) characteristics sufficiently described?** | **5. If interventional and random allocation was possible, was it described?** | **6. If interventional and blinding of investigators was possible, was it reported?** | **7. If interventional and blinding of subjects was possible, was it reported?** | **8. Outcome and (if applicable) exposure measure(s) well defined and robust to measurement / misclassification bias? means of assessment reported?** | **9. Sample size appropriate?** | **10. Analytic methods described / justified and appropriate?** | **11. Some estimate of variance is reported for the main results?** | **12. Controlled for confounding?** | **13. Results reported in sufficient detail?** | **14. Conclusions supported by the results?** | **Total**  **(%)** |
| --- | --- | --- | --- | --- | --- | --- | --- | --- | --- | --- | --- | --- | --- | --- | --- |
| Daly et al 1999 | 2 | 2 | 2 | 2 | NA | NA | NA | 2 | 2 | 2 | 2 | 0 | 2 | 2 | 90 |
| **Qualitative** | **1. Question /objective clearly described?** | **2. Design evident and appropriate to answer study question?** | **3. Context for the study is clear?** | **4. Connection to a theoretical framework / wider body of knowledge?** | **5. Sampling strategy described, relevant and justified?** | **6. Data collection methods clearly described and systematic?** | **7. Data analysis clearly described, complete and systematic?** | **8. Use of verification procedure(s) to establish credibility of the study?** | **9. Conclusion supported by the results?** | **10. Reflexivity of the account?** |  |  |  |  | **Total (%)** |
| Mendes et al 2010 | 2 | 2 | 2 | 1 | 2 | 2 | 2 | 0 | 2 | 0 |  |  |  |  | 75 |
| Mendes et al, 2015 | 2 | 2 | 2 | 2 | 2 | 2 | 2 | 2 | 2 | 1 |  |  |  |  | 95 |
| Peters et al, 2004 | 2 | 2 | 2 | 2 | 2 | 2 | 2 | 1 | 2 | 0 |  |  |  |  | 85 |
| Peters et al, 2006 | 2 | 2 | 2 | 2 | 2 | 2 | 2 | 2 | 2 | 1 |  |  |  |  | 95 |
| Peters et al, 2012 | 2 | 2 | 2 | 2 | 2 | 2 | 2 | 2 | 2 | 0 |  |  |  |  | 90 |
| McLeod et al, 2018 | 2 | 2 | 2 | 2 | 2 | 2 | 1 | 1 | 2 | 0 |  |  |  |  | 80 |
| Spiers et al, 2020 | 2 | 2 | 2 | 2 | 2 | 2 | 2 | 2 | 2 | 0 |  |  |  |  | 90 |
| Stopford et al, 2020 | 2 | 2 | 2 | 2 | 2 | 2 | 2 | 2 | 2 | 0 |  |  |  |  | 90 |
| Average |  |  |  |  |  |  |  |  |  |  |  |  |  |  | **88** |

The above criteria were scored either a “2” for yes, “1” for partial or “0” for no, as per guidelines provided by the QualSyst Tool [Kmet et al, 2004]. NA; not available.
